# The glomerular parietal epithelial cell’s responses are influenced by SM22 alpha levels

**DOI:** 10.1186/1471-2369-15-174

**Published:** 2014-11-06

**Authors:** Shokichi Naito, Jeffrey W Pippin, Stuart J Shankland

**Affiliations:** Division of Nephrology Department of Medicine, University of Washington School of Medicine, Box 356521, 1959 NE Pacific St., Seattle, WA 98195-6521 USA; Department of Nephrology, Kitasato University School of Medicine, 1-15-1 Kitasato, Minami-ku, Sagamihara, Kanagawa 252-0329 Japan

**Keywords:** Regeneration, WT-1, Podocyte, Glomerulus, Progenitor

## Abstract

**Background:**

Studies have shown in several diseases initially affecting podocytes, that the neighboring glomerular parietal epithelial cells (PECs) are secondarily involved. The PEC response might be reparative under certain circumstances, yet injurious under others. The factors governing these are not well understood. We have shown that SM22α, an actin-binding protein considered a marker of smooth muscle differentiation, is upregulated in podocytes and PECs in several models of podocyte disease. However, the impact of SM22α levels on PECs is not known.

**Methods:**

Experimental glomerular disease, characterized by primary podocyte injury, was induced in aged-matched SM22α +/+ and SM22α -/- mice by intraperitoneal injection of sheep anti-rabbit glomeruli antibody. Immunostaining methods were employed on days 7 and 14 of disease.

**Results:**

The number of PEC transition cells, defined as cells co-expressing a PEC protein (PAX2) and podocyte protein (Synaptopodin) was higher in diseased SM22α -/- mice compared with SM22α +/+ mice. WT1 staining along Bowman’s capsule is higher in diseased SM22α -/- mice. This was accompanied by increased PEC proliferation (measured by ki-67 staining), and an increase in immunostaining for the progenitor marker NCAM, in a subpopulation of PECs in diseased SM22α -/- mice. In addition, immunostaining for vimentin and alpha smooth muscle actin, markers of epithelial-to-mesenchymal transition (EMT), was lower in diseased SM22α -/- mice compared to diseased SM22α+/+ mice.

**Conclusion:**

SM22α levels may impact how PECs respond following a primary podocyte injury in experimental glomerular disease. Absent/lower levels favor an increase in PEC transition cells and PECs expressing a progenitor marker, and a lower EMT rate compared to SM22α +/+ mice, where SM22 levels are markedly increased in PECs.

## Background

Adult podocytes are terminally differentiated glomerular epithelial cells that are unable to proliferate adequately to replace those lost in glomerular diseases [1, 2]. Reduced podocyte number leads to proteinuria and glomerulosclerosis in diabetic and non-diabetic glomerular diseases [2–7]. The interplay between the parietal epithelial cell (PECs) and visceral epithelial cell (podocyte) has been keenly studied recently in experimental and human glomerular diseases. Studies in humans, and in adolescent PEC reporter mice, support a paradigm where PECs function as local progenitors for podocytes [8–10]. In normal human and rodent glomeruli, a subset of PECs co-express proteins considered unique to both podocytes, and to PECs [8, 9, 11–14]. Some have called these transitional cells [8, 11–14]. Moreover, a subset of PECs in humans and rodents express proteins considered as general markers for stem/progenitor cells, suggesting the possibility that a renal progenitor system exists [15]. Recent studies in mice have disputed this concept [16–19].

Two lines of evidence suggest that rather than PECs being regenerative, PECs augment glomerular damage following podocyte injury. First, their activation as evidenced by the de novo expression of CD44 likely contributes to disease progression by augmenting scarring and crescent formation under certain circumstances [20, 21]. Second, in response to injury PECs can also undergo epithelial-to-mesenchymal transition (EMT) [22–27], which is a phenotypic change characterized by loss/decrease of epithelial characteristics while attaining features of mesenchymal cells. Another view is that these different PEC “phenotypes” (i.e. progenitors, activated-CD44 positive, EMT) are not mutually exclusive.

The mechanisms associated with PEC EMT are not well defined. SM22α, also known as transgelin, is a 22-kDa actin-binding protein of the calponin family, a cytoskeleton associated protein and one of the earliest markers of smooth muscle differentiation [28, 29]. Although SM22α is absent in normal glomeruli, it is markedly increased in diseased glomeruli, in both podocytes and PECs [30–32]. The kidneys of SM22α-deficient mice develop normally and appear similar to wildtype mice histologically [33]. However, we and others have shown that SM22α staining is markedly increased in experimental animal models of membranous nephropathy (PHN model), FSGS (PAN, ADR nephropathy, obesity-related glomerulopathy), crescentic glomerulonephritis (anti-GBM nephritis model), mesangial proliferation (anti-Thy1 model) and obstructive nephropathy (UUO model) [27, 30, 31, 34–39]. In these studies, de novo SM22α expression was detected in podocytes, as well as in PECs. When experimental crescentic glomerulonephritis was induced, SM22α+/+ mice had more severe glomerular disease compared to SM22α -/- mice, marked by greater podocyte apoptosis, lower podocyte number, more proliferation, and increased activation of Erk1/2 [32], indicating SM22α likely plays a deleterious role in podocytes.

The focus of these earlier studies was on SM22α and the podocyte [32, 34–36]. Since then, interest in PECs has increased substantially, enhancing our understanding of this less well defined glomerular epithelial cell [21, 22, 40, 41]. Accordingly, in the current experiments, we studied the potential biological affect of SM22α on PECs in experimental glomerular disease, with a focus on glomerular epithelial transition cells and EMT.

## Methods

### Passive nephrotoxic models of experimental crescentic glomerulonephritis

SM-CreERT2(ki) transgenic mice were used, which have been extensively characterized previously [32]. We have also previously reported that giving these mice sheep anti-glomerular antibody used in this study leads to passive nephrotoxic crescentic glomerulonephritis, with varying degrees of capillary loop dilatation, mesangial expansion, crescent formation with increased WT-1 and Ki-67 staining in the glomerular tuft [32, 42]. Please not that this is not the same antibody used to induce an experimental model of classic FSGS, which we have reported is characterized by reduced podocyte number and an absence of glomerular epithelial cell proliferation [11, 13, 14, 43].

The passive nephrotoxic nephritis model was induced in male SM22α wildtype (+/+) and null (-/-) mice, aged 12 wk, by intraperitoneal injection of sheep anti-rabbit glomeruli antibody [12. 5 mg/20 g body weight 2 doses at days 0 and 3]. These mice were randomly assigned into the following groups: day 0 (n = 6 +/+ and -/- mice), day 7 (n = 10, +/+ and -/- mice) and day 14 (n = 5 +/+ and -/- mice). The sheep anti-rabbit glomeruli antibody was produced by immunizing sheep with whole rabbit glomeruli, as previously described [42]. Control animals did not receive the sheep anti-rabbit glomeruli antibody. The animal care committee of the University of Washington, School of Medicine reviewed and approved the experimental protocol and animal procedures were conducted following Institutional Animal Care and Use Committee review.

### Immunohistochemistry staining methods

#### Identifying transition cells

To identify and quantitate the number of glomerular epithelial cells that express both a podocyte and PEC proteins (called glomerular epithelial transition cells), double immunostaining was performed as follows. Indirect immunofluorescence was performed on 4 um-thick section kidney biopsies fixed in formalin and embedded in paraffin as we have previously reported [12–14, 32, 43]. In brief, paraffin was removed using Histoclear (National Diagnostics, Atlanta, Ga., USA), and sections were rehydrated in ethanol. Antigen retrieval was performed by boiling sections in the microwave in 1 mM EDTA, pH 6.0. Nonspecific protein binding was blocked with background buster (Accurate Chemical & Scientific, Westbury, NY, USA) and endogenous biotin activity was quenched with the Avidin/biotin blocking kit (Vector Laboratories, Burlingame, CA, USA). After the blocking steps described above, tissue sections were incubated overnight at 4°C with the primary antibodies. The following primary antibodies were used: rabbit anti-rat paired box gene 2 (PAX2, a PEC nuclear protein) polyclonal antibody diluted 1:500 (Zymed Laboratories, South San Francisco, Calif., USA) and mouse anti- Synaptopodin (Synpo, a podocyte protein) monoclonal antibody, diluted 1:10 (Fitzgerald). The appropriate biotinylated secondary antibody (Vector Laboratories) was applied followed by Streptavidin, AlexaFluor 594 conjugate and Alexa 488 conjugate.

### Identifying podocyte and potential PEC progenitors

The rabbit anti-WT-1 polyclonal antibody (Santa Cruz Biotechnology, USA), and NCAM ( Millipore, MA, USA) were used to identify podocyte and potential PEC progenitors . Positive cells being a bluish-gray color were visualized with the Vector SG substrate kit (Vector).

### Identifying EMT

Indirect immunoperoxidase immunostaining was performed for PAX2 as described above, in combination with vimentin (1:100 Santa Cruz, CA, USA) and alpha-smooth muscle actin (α-SMA, 1:400 Abcam, UK).). Omission of the primary antibody was used as a negative control. Vimentin and α-SMA staining were visualized with the Vector SG substrate kit; positive cells are bluish-gray color (Vector). Blocking steps were performed following SG substrate color development. Because α-SMA and PAX2 antibody were developed in rabbits, an anti-rabbit IgG antibody Fab fragment (Jackson ImmunoResearch, West Grove, PA, USA) was used to saturate all the binding sites created during the first set of staining. In addition, peroxidase activity derived from the first set of staining was also blocked using alkaline phosphatase/horseradish peroxidase block (SurModics, MN, USA). Next, a second set of staining was performed for PAX2. A rabbit-on-rodent AP-polymer kit (Biocare Medical, CA, USA) was used for additional blocking and substitutive secondary antibody according to the manufacturer`s protocol. Staining was visualized with the Warp Red chromogen kit, red color (Biocare Medical).

### Measuring proliferation

To quantitate changes in PEC proliferation in SM22α mice rabbit anti-Ki-67 monoclonal antibody (Thermo Fisher Scientific, Fremont, Calif., USA) were used to identify proliferating cells. Both of them were visualized with the Vector SG substrate kit, with positive cells being a bluish-gray color (Vector). For all stains, negative controls consisted of omitting the primary antibodies.

### Staining quantitation and statistical analysis

Quantification of positively stained cells was performed on individual animals at each time point using a combination of bright-field and fluorescent microscopy as we have reported [12–14]. The mean number of glomeruli analyzed was 88 (95% CI: 82–94) per animal. Because of known changes in glomerular size with aging and weight increasing, ImageJ software was used to measure the length of the Bowman’s basement membrane and glomerular tuft area according to ‘The ImageJ User Guide’ (version 1.44) as we described previously [12–14]. These measures were then used as denominators for the number of positively stained podocytes, and double-stained cells along Bowman’s capsule and in tuft, respectively.

Oneway ANOVA with unpaired t-test was calculated and a p-value below 0.05 was considered significant. The quantitative analyses of Bowman’s capsule length and tuft area were evaluated by ImageJ. All values are means +/- SEM. Statistical significance was evaluated using StatFlex version 6 (Artech Co., Ltd., Osaka, Japan).

## Results

### PEC number is higher in SM22 null mice with experimental glomerular disease compared to SM22 wildtype mice

**Figure 1:**
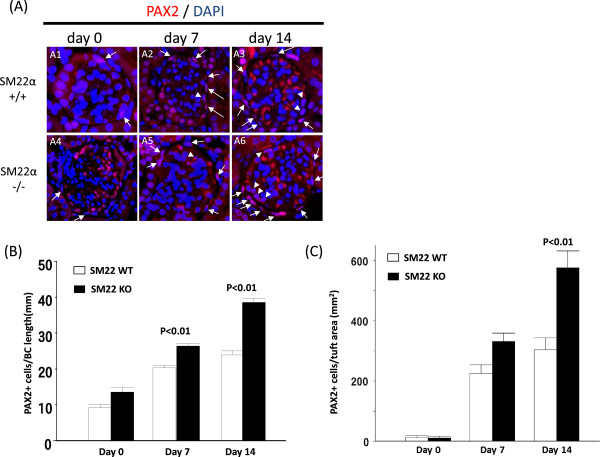
**PECs number in SM22 null mice is higher with experimental glomerular disease compared to wildtypes. A)** Representative images of PAX2 staining (red) at × 630 original magnification. SM22α +/+ is A1-A3. SM22α -/- is A4-A6. A1, A4: day 0. A2, A5: day 7. A3, A6: day 14. PECs are on Bowman’s capsule (arrow) and in tuft (arrow head). **B)** Number of PAX2 positive cell along BBM / Bowman’s capsule (BC) length (mm) is increased in SM22α -/- mice, compared with SM22α +/+ mice. **C)** Number of PAX2 positive cells in tuft area / tuft area (mm^2^) is increased in SM22α -/+ - mice, compared with SM22α +/+ mice.

To determine PEC number, PAX2 staining was performed and quantitated (Figure 1). Because of potential changes in glomerular size during disease, the length of Bowman’s capsule, measured in millimeters, was used as the denominator. PEC number was expressed as the number of PAX2 positive cells/Bowman’s capsule length in millimeters (Figure 1B). There was no statistical significance difference in PEC number at baseline between normal SM22α +/+ and SM22α -/- mice (9.19 ± 0.93 vs. 13.58 ± 1.26 PAX2 positive cells per mm of Bowman’s capsule; P =0.08). However, in experimental glomerular disease, PEC number was higher than baseline, and SM22α -/- mice had a higher number of PECs compared to SM22α +/+ mice at day 7 (20.33 ± 0.70 vs. 26.38 ± 0.73 PAX2+ cells/Bowman’s capsule length in mm, P <0.01), and at day 14 (23.92 ± 1.14 vs. 38.55 ± 1.18; P <0.01) (Figure 1B).

Because staining for PEC proteins have been detected in cells within the glomerular tuft, [8, 9, 12–14], the number of PAX2 positive cells was also measured in the tuft, expressed per mm^2^ area. Very few PAX2 positive cells were in the tuft at baseline in SM22α +/+ and SM22α -/- mice (12.66 ± 6.24 PAX2+ cells/mm^2^ of glomerular tuft vs.10.08 ± 6.60; P = 1.00). However, in experimental glomerular disease, the number of PAX2 positive cells in the tuft increased, but was significantly higher in SM22α -/- mice compared to SM22α +/+ mice at day 14 (303.13 ± 41.07 vs. 576.51 ± 55.52; P <0.01) (Figure 1C). The trend was also higher in SM22 -/- mice at day 7, but did not reach statistical significance (225.27 ± 29.00vs. 331.59 ± 27.79; P =0.126).

### Transition cells in SM22 null mice are higher with experimental glomerular disease compared to wildtypes

**Figure 2:**
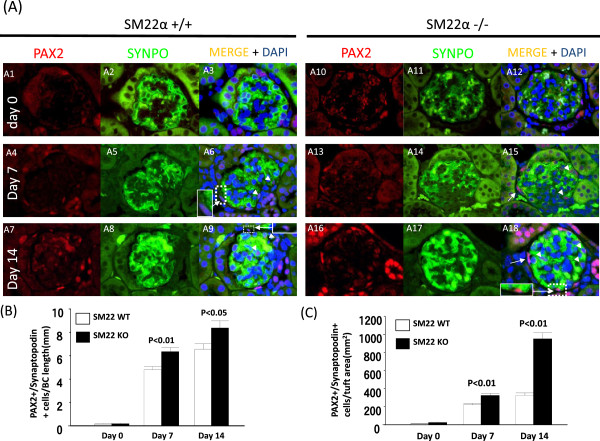
**Transition cells in SM22 null mice are higher with experimental glomerular disease compared to wildtypes. A)** Representative images of transition cells at × 630 original magnification. Glomerular epithelial transition cells defined PAX2 (red, nuclear) and SYNPO (green, cytoplasmic) double positive cells. SM22α +/+ is A1-A9. SM22α -/- is A10-A18. Day 0 (A1-A3, A10-A12), day 7 (A4-A6, A13-A15), day 14 (A7-A9, A16-A18) were reacted anti PAX2 antibody (Ab) (A1, A4, A7, A10, A13, A16) and anti Synaptopodin (SYNPO) Ab (A2, A5, A8, A11, A14, A17). The images were merged (A3, A6, A9, A12, A15, A18). The glomerular transition cells are on Bowman’s capsule (arrow) and in tuft (arrow head). **B)** Number of glomerular epithelial transition cells along BBM / Bowman’s capsule (BC) length (mm) is increased in SM22α -/- mice, compared with SM22α +/+ mice. **C)** Number of glomerular epithelial transition cells in tuft area / tuft area (μm^2^) is increased in SM22α -/+ - mice, compared with SM22α +/+ mice.

Double immunofluorescent staining for PAX2 (PEC marker) and synaptopodin (podocyte marker) was performed to determine the number of glomerular epithelial transition cells along Bowman’s capsule, and within the glomerular tuft (Figure 2). At baseline, very few PAX2^+^/Synaptopodin^+^ cells were detected along Bowman’s capsule in normal SM22α +/+ mice or -/- mice (0.15 ± 0.04 vs. 0.18 ± 0.05 PAX2^+^/Synaptopodin^+^ cells/mm of Bowman’s capsule, P =1.00). The number of transition cells along Bowman’s capsule increased significantly in both strains with experimental glomerular disease, although the magnitude of increase was greater in SM22α -/- mice at day 7 (6.35 ± 0.34 vs. 4.79 ± 0.28 PAX2^+^/Synaptopodin^+^ cells/mm of Bowman’s capsule, P <0.01 vs. +/+), and day 14 (8.38 ± 0.60 vs. 6.56 ± 0.46, P <0.05 vs. +/+) (Figure 2B).

Transition cells that were barely detected in the glomerular tuft at baseline in SM22α +/+ or -/- mice (13.19 ± 3.09, vs. 23.53 ± 5.95 PAX2^+^/Synaptopodin^+^ cells/mm^2^ of glomerular tuft; P = 1.00 ), increased in glomerular disease. The number of transition cells increased in SM22α -/- mice with disease at day 7 (324.89 ± 21.20 vs. 219.82 ± 16.07, P <0.01 vs. +/+), and at day 14 (951.32 ± 67.17 vs. 326.41 ± 26.33, P <0.01 vs. +/+) (Figure 2C).

These data show that the number of glomerular transition cells along Bowman’s capsule and in the glomerular tuft was higher in diseased SM22 -/- mice compared to diseased SM22 +/+ mice.

### NCAM staining in SM22 null mice is higher with experimental glomerular disease compared to wildtypes

**Figure 3:**
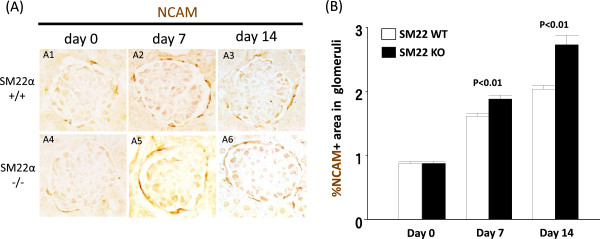
**NCAM staining in SM22 null mice is higher with experimental glomerular disease compared to wildtypes. A)** Representative images of NCAM staining (brown) at × 630 original magnification. SM22α +/+ is A1-A3. SM22α -/- is A4-A5. A, D: day 0. B, E: day 7. C, F: day 14. **B)** % of NCAM positive area in glomeruli is increased in SM22α -/- mice, compared with SM22α +/+ mice.

Neural cell adhesion molecule (NCAM) staining was used to identify potential PEC progenitors, similar to that described by Benigni (Figure 3A) [44]. At baseline, occasional NCAM staining was detected in cells lining Bowman’s capsule in normal SM22α +/+ and -/- mice (0.87 ± 0.03 vs. 0.88 ± 0.03; P =1.00). NCAM staining increased in glomerular disease, but was higher in SM22 -/- mice at day 7 (1.88 ± 0.06 vs. 1.61 ± 0.05, P <0.01 vs. +/+) and at day 14 (2.73 ± 0.14 vs. 2.03 ± 0.06,. P <0.01 vs. +/+) (Figure 3B). NCAM staining localized to PEC predominantly.

### WT1 staining along Bowman’s capsule is higher in SM22 null mice with disease

**Figure 4:**
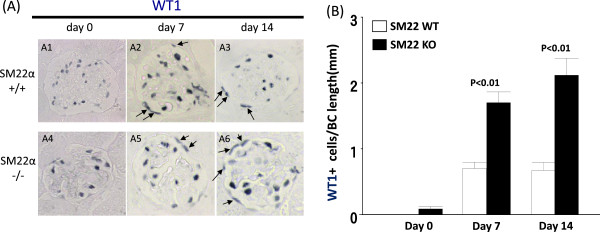
**WT1 staining along Bowman’s capsule in SM22α -/- mice is higher with experimental glomerular disease. A)** Representative images of WT1 staining (blue/gray) at × 630 original magnification. SM22α +/+ is A1-A3. SM22α -/- is A4-A6. A1, A4: day 0. A2, A5: day 7. A3, A6: day 14. Allows are WT1 positive cells. **B)** Number of WT1 positive cells along BBM / Bowman’s capsule (BC) length (mm) is increased in SM22α -/- mice, compared with SM22α +/+ mice.

WT-1 staining has traditionally been used as a marker to identify podocytes [45], WT-1 staining was barely detected along Bowman’s capsule in SM22α +/+ and -/- mice at baseline (0.00 ± 0.00 vs. 0.09 ± 0.04; P = 0.52) (Figure 4). However, in experimental glomerular disease, the number of WT-1 positive cells along Bowman’s capsule was higher in SM22α -/- mice at day 7 (1.70 ± 0.16 vs. 0.70 ± 0.09, P <0.01 vs. +/+), and at day 14 (2.12 ± 0.26 vs. 0.67 ± 0.12, P <0.01 vs. +/+). Thus, similar to other studies [9, 11–14, 40, 46] WT-1 staining is increased in cells lining Bowman’s capsule in mice with disease, and is higher SM22 -/- mice.

### Proliferation is higher in diseased SM22 null mice

**Figure 5:**
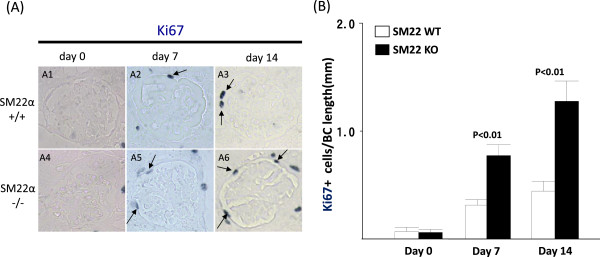
**Proliferation is higher along Bowman’s capsule in experimental glomerular disease in SM22 null mice. A)** Immunohistochemistry of Ki67 (blue/gray). SM22α +/+ is A1-A3. SM22α -/- is A4-A6. A1, A4: day 0. A2, A5: day 7. A3, A6: day 14. **B)** Number of Ki67 positive cells along BBM / Bowman’s capsule (BC) length (mm) is increased in SM22α -/- mice, compared with SM22α +/+ mice.

Ki-67 immunostaining, a proliferation marker [47], was barely detected along the Bowman’s capsule at baseline in SM22α +/+ and -/- mice (0.07 ± 0.04 vs. 0.06 ± 0.03 positive cells/Bowman’s capsule length; P = 1.00) (Figure 5A1 & A4). Although the number increased in diseased SM22α +/+ mice, the number of Ki-67 cells along the Bowman’s capsule was higher in diseased SM22α -/- mice at day 7 (0.31 ± 0.05 vs. 0.77 ± 0.10; P <0.01), and at day 14 (0.44 ± 0.09 vs. 1.28 ± 0.19; P <0.01) (Figure 5A2, A3, A5, A6). These data show that a subset of cells along Bowman’s capsule proliferate in disease, more so in SM22α -/- mice than SM22α +/+ mice (Figure 5B).

**Figure 6:**
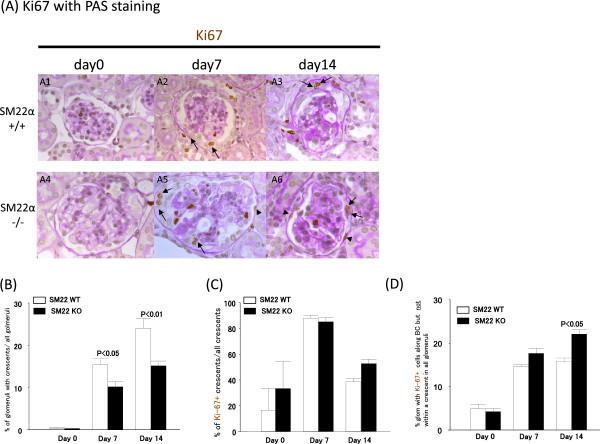
**The crescent is higher in diseased SM22α +/+ mice. A)** Representative images of Ki67 staining (blue/gray) at × 630 original magnification. SM22α +/+ is A1-A3. SM22α -/- is A4-A6. A1, A4: day 0. A2, A5: day 7. A3, A6: day 14. Allows are Ki67+ cells in crescents. Allow heads are Ki67+ cells on Bowman’s capsule (BC). **B)** % glomeruli of crescent. **C)** % crescent within Ki-67+ cells / all crescents. **D)** % glomeruli with Ki67+ cells outside of crescents on Bowman’s capsule (BC) / all glomeruli.

Double staining for Ki-67 and PAS was performed to evaluate proliferating cells in crescents (Figure 6A). In SM22α +/+ mice at day 7, 15.36 ± 1.46% of glomeruli contained crescents. SM22α -/- mice with disease had fewer crescents at day 7 (10.17 ± 1.19% vs. 15.36 ± 1.46%; P <0.05 vs. +/+) (Figure 6B). The percentage of crescents with Ki67+ demonstrate no significant difference between SM22α +/+ and SM22α -/- mice at day 7(87.93 ± 6.75% vs. 85.15 ± 10.24%, P = 1.00), and at day 14 (38.50 ± 15.02% vs. 52.69 ± 7.50%, P = 0.15). Ki-67 staining was used as a marker of cell proliferation (Figure 6C). Similar to the results shown above, the percentage of glomeruli with cells staining for Ki67 that were along Bowman’s capsule but not within a crescent was higher in SM22α -/- compared to SM22α +/+ at day 7 (17.66 ± 1.12 vs. 14.65 ± 0.43; P = 0.32) and at day 14 (22.1 ± 1.00 vs. 15.83 ± 0.79, P <0.05) (Figure 6D).

### Epithelial-to-mesenchymal transformation of PECs is lower in disease in SM22 null mice with experimental glomerular

**Figure 7:**
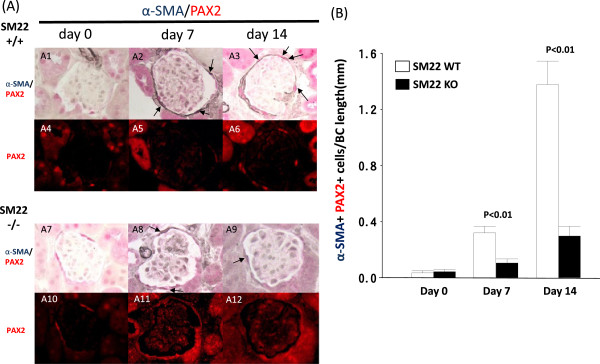
**Double positive cells for PAX2 and α-SMA at × 630 original magnification. A)** Double positive cells for PAX2 (red, nuclear) and α-SMA (blue/gray, cytoplasmic) at × 630 original magnification. A1-A3: representative images of PAX2/α-SMA double staining at × 630 original magnification in SM22α +/+ mice. A4-A6: fluorescent microscopic view of the A1-A3 bright-field view, where only PAX2 staining is seen because only the warp-red substrate is visible by fluorescent microscopy. A7-A9: representative images of PAX2/α-SMA double staining at × 630 original magnification in SM22α -/- mice. A10-A12: fluorescent microscopic view of the A7-9 bright-field view, where only PAX2 staining is seen because only the warp-red substrate is visible by fluorescent microscopy. A1, A4, A7, A10: day 0. A2, A5, A8, A11: day 7. A3, A6, A9, A12: day 14. **B)** Number of double positive cells for PAX2 and α-SMA along BBM/Bowman’s capsule (BC) length (mm) is increased in SM22α -/- mice, compared with SM22α +/+ mice.

Epithelial-to-mesenchymal transformation (EMT) has been reported in PECs in disease, and correlates with worse outcomes [25, 48]. In order to determine if the levels of SM22α affected PEC EMT, PAX2 (used as a PEC marker) was double-stained with classic EMT markers α-smooth muscle actin (α-SMA) or vimentin (Figure 7). α-SMA^+^PAX2^+^ double positive cells were very occasionally detected along Bowman’s capsule in SM22α +/+ and SM22α -/- mice at baseline (0.03 ± 0.02 vs. 0.04 ± 0.02; P = 1.00). At day 7, the number of α-SMA^+^PAX2^+^ double positive cells was higher in SM22+/+ mice (0.32 ± 0.05 vs. 0.10 ± 0.03; P = <0.01 vs. -/-). The number of α-SMA^+^PAX2^+^ double positive cells was even higher at day 14 in SM22α +/+ mice compared to SM22α -/- mice (1.38 ± 0.17 vs. 0.30 ± 0.07; P <0.01 vs. -/-) (Figure 7).

**Figure 8:**
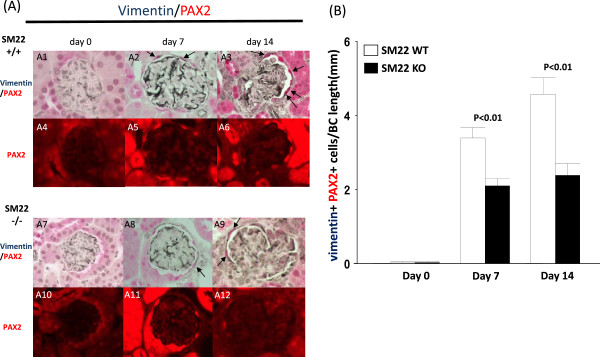
**Double positive cells for PAX2 (red) and vimentin at × 630 original magnification. A)** Double positive cells for PAX2 (red, nuclear) and vimentin (blue gray, cytoplasmic) at × 630 original magnification. A1-A3: representative images of PAX2/vimentin double staining at × 630 original magnification in SM22α +/+ mice. A4-A6: fluorescent microscopic view of the A1-A3 bright-field view, where only PAX2 staining is seen because only the warp-red substrate is visible by fluorescent microscopy. A7-A9: representative images of PAX2/vimentin double staining at × 630 original magnification in SM22α -/- mice. A10-A12: fluorescent microscopic view of the A7-A9 bright-field view, where only PAX2 staining is seen because only the warp-red substrate is visible by fluorescent microscopy. A1, A4, A7, A10: day0. A2, A5, A8, A11: day 7. A3, A6, A9, A12: day 14. **B)** Number of double positive cells for PAX2 and vimentin along BBM / Bowman’s capsule (BC) length (mm) is increased in SM22α -/- mice, compared with SM22α +/+ mice.

Double staining for vimentin and PAX2, was barely detected along Bowman’s capsule at baseline in SM22α +/+ and -/- mice (0.03 ± 0.02 vs. 0.03 ± 0.02; P =1.00). The number of vimentin^+^PAX2^+^ double positive cells along the Bowman’s capsule was higher in SM22α +/+ mice at day 7 (2.10 ± 0.20 vs. 3.39 ± 0.27; P <0.01 vs. -/-) and day 14 (2.38 ± 0.32 vs. 4.57 ± 0.44; P <0.01 vs. -/-) (Figure 8).

These data show that two markers of EMT are significantly higher in PECs in SM22 wildtype mice compared to null mice with disease.

## Discussion

Studies in man show that PECs might play a critical role in glomerular repair through their progenitor function [8, 9, 15, 21]. However, PECs might paradoxically contribute to the deterioration of glomerular function by augmenting scarring and crescent formation under certain circumstances in man and mouse [21]. The precise mechanisms underlying this dichotomy are poorly understood. Because SM22α was markedly increased in PECs (and podocytes) in diseases considered primarily podocyte in nature [27, 30, 31, 34–39], the purpose of the current studies was to determine if the levels of SM22 had any affect on certain PEC properties in response to primary injury to podocytes.

The first major finding was that in experimental glomerular disease, there was a direct association between reduced SM22 levels and increased PEC number, including an increase in the PEC transition cell subpopulation. The latter was supported by a higher number of cells co-staining for a PEC protein, and two podocyte proteins, namely synapotopodin and WT-1. Moreover, there is a subset of PECs along Bowman’s capsule that have increased expression for NCAM. NCAM expression has been shown in metanephric mesenchyme and in the glomerular capsule in the mature kidney [49, 50]. Others have shown that NCAM is a marker of renal progenitor cells [44, 51]. This suggests, but does not prove, that the subpopulation of PECs that might acquire a progenitor phenotype in disease is higher in the absence of SM22. Given that studies have shown that reduced SM22α levels have a proliferative effect in vascular smooth muscle cells and prostate tumor cells [52, 53], and that SM22 levels were significantly reduced in proliferating cells in prostate, lung, breast, gliobrastoma and colorectal cancers [54–56], it is perhaps not surprising that PECs had higher proliferation in mice deficient in SM22α.

A second major finding in the current study was that although epithelial-to-mesenchymal transition (EMT) occurred in both strains of mice with disease, the magnitude of EMT was higher in SM22 +/+mice compared to SM22 -/- mice, judged by the EMT markers alpha smooth muscle actin and vimentin. EMT in kidney and non-kidney cell types has been associated with fibrosis, and is therefore considered undesirable [57]. Indeed, studies have previously demonstrated EMT in PECs. Studies by Yee-Yung [23] and Shimizu [58] showed that PEC EMT was deleterious. In contrast, Swetha showed that EMT plays a role in plasticity of cultured PECs [26], which is considered beneficial. In the current study our data supports a notion that PEC EMT is significantly lower in mice lacking SM22α. One might the current data that the lower PEC EMT in null mice with disease is consistent with an overall improved outcome.

Several limitations to this manuscript are noted, although they do not substantially impact the enthusiasm for the findings. First, although mice with genetically altered SM22 level are used, this is largely descriptive. Second, an alternate explanation to both major findings is that they are independent from SM22 levels, because the podocyte lesion is worse in null mice. Regardless, this is informative, because the latter explanation would suggest that the degree of PEC response correlates with the degree of podocyte damage. One might even argue that the differences in the PEC responses are due to differences in the immune response to injury between the null and wildtype strains used. Please note that although the data was not shown, there were no differences between the two mouse strains in the amount of disease-inducing antibody deposited in podocytes (it does not deposit in PECs). We have previously reported that this model is not characterized by an inflammatory cell response, which makes this an unlikely contributor to underlie any differences between SM22 -/- and +/+ mice.

## Conclusions

PECs are increasingly being recognized as second responders following a primary injury to neighboring podocytes. On one hand, the secondary response by PECs might be reparative if they indeed are a source of podocyte progenitors. The data from the current study suggests that they do express a putative progenitor marker (NCAM), and that the number of transition cells is increased. These events are augmented in the absence of SM22α. On the other hand, the secondary response by PECs can be deleterious if they are activated to express CD44 or undergo EMT. Our data shows that PEC EMT is reduced in mice lacking SM22α. Taken together, the current data support a model that when PECs are unable to express (or increase) SM22α in a model of podocyte injury, they tend to favor a less injurious response judged by enhanced progenitor markers and less EMT. Further studies are needed to better delineate these pathways and to provide functional proof of this concept.

## Abbreviations

PEC: Parietal epithelial cell
FSGS: Focal segmental glomerulosclerosis
EMT: Epithelial-to-mesenchymal transformation
α-SMA: Alpha-smooth muscle actin.
